# Eukaryotic initiation factor 4E-binding protein as an oncogene in breast cancer

**DOI:** 10.1186/s12885-019-5667-4

**Published:** 2019-05-23

**Authors:** Alexandria C. Rutkovsky, Elizabeth S. Yeh, Stephen T. Guest, Victoria J. Findlay, Robin C. Muise-Helmericks, Kent Armeson, Stephen P. Ethier

**Affiliations:** 1 Department of Pathology and Laboratory Medicine, Medical University of South Carolina, 171 Ashley Avenue, MSC 908, Charleston, SC 29425 USA; 2 Department of Cell and Molecular Pharmacology and Experimental Therapeutics, Medical University of South Carolina, 173 Ashley Avenue, BSB 358, MSC 509, Charleston, SC 29425 USA; 3 Hollings Cancer Center, Medical University of South Carolina, 86 Jonathan Lucas Street, Charleston, SC 29425 USA; 4 Department of Computational Medicine and Bioinformatics, University of Michigan, 500 S. State Street, Ann Arbor, MI 48109 USA; 5 Department of Regenerative Medicine and Cell Biology, Medical University of South Carolina, 173 Ashley Avenue, BSB 601, MSC 508, Charleston, SC 29425 USA; 6 Department of Public Health Sciences, Medical University of South Carolina, 135 Cannon Street Suite 303 MSC 835, Charleston, USA

**Keywords:** EIF4EBP1, 4EBP1, 4E-BP1, PHAS-I, 8p11–12, 8p12–11, 8p11-p12, Chromosomal abnormality, Oncogene, Amplification, Driver, Breast cancer, Estrogen receptor

## Abstract

**Background:**

Eukaryotic Initiation Factor 4E-Binding Protein (*EIF4EBP1*, 4EBP1) is overexpressed in many human cancers including breast cancer, yet the role of 4EBP1 in breast cancer remains understudied. Despite the known role of 4EBP1 as a negative regulator of cap-dependent protein translation, 4EBP1 is predicted to be an essential driving oncogene in many cancer cell lines in vitro, and can act as a driver of cancer cell proliferation. *EIF4EBP1* is located within the 8p11-p12 genomic locus, which is frequently amplified in breast cancer and is known to predict poor prognosis and resistance to endocrine therapy.

**Methods:**

Here we evaluated the effect of 4EBP1 targeting using shRNA knock-down of expression of 4EBP1, as well as response to the mTORC targeted drug everolimus in cell lines representing different breast cancer subtypes, including breast cancer cells with the 8p11-p12 amplicon, to better define a context and mechanism for oncogenic 4EBP1.

**Results:**

Using a genome-scale shRNA screen on the SUM panel of breast cancer cell lines, we found 4EBP1 to be a strong hit in the 8p11 amplified SUM-44 cells, which have amplification and overexpression of 4EBP1. We then found that knock-down of 4EBP1 resulted in dramatic reductions in cell proliferation in 8p11 amplified breast cancer cells as well as in other luminal breast cancer cell lines, but had little or no effect on the proliferation of immortalized but non-tumorigenic human mammary epithelial cells. Kaplan-Meier analysis of *EIF4EBP1* expression in breast cancer patients demonstrated that overexpression of this gene was associated with reduced relapse free patient survival across all breast tumor subtypes.

**Conclusions:**

These results are consistent with an oncogenic role of 4EBP1 in luminal breast cancer and suggests a role for this protein in cell proliferation distinct from its more well-known role as a regulator of cap-dependent translation.

**Electronic supplementary material:**

The online version of this article (10.1186/s12885-019-5667-4) contains supplementary material, which is available to authorized users.

## Background

Estrogen Receptor-positive (ER+) breast cancer accounts for ~ 70% of all breast cancers. Currently, this subtype of breast cancer is treated with endocrine therapy as the standard of care. However, resistance to endocrine therapy is a significant clinical problem and is a leading cause of breast cancer mortality. Amplification of the 8p11-p12 region of the human genome, which occurs in ~ 20–30% of metastatic ER+ breast cancers, is associated with resistance to endocrine therapy and poor prognosis [1].

Our laboratory and others have demonstrated the importance of the 8p11-p12 amplicon and many of its genes in the development and pathogenesis of breast cancer [2–33], including its role in endocrine resistance. The amplicon is composed of four distinct regions, designated A1-A4, each of which contains a number of overexpressed genes [5, 11]. At least 11 genes are associated with the A2 region of the amplicon [5]. The Eukaryotic Initiation Factor 4E-Binding Protein (*EIF4EBP1*) sequence is located on the short arm of chromosome 8: 38,030,502–38,060,365 (GRCh38.p7; current assembly) and is amplified along with other A1 and A2 region genes. The protein product (herein referred to as 4EBP1) encoded by *EIF4EBP1* is canonically regarded as a translational repressor protein that interacts with eukaryotic initiation factor 4E (eIF4E) and represses translation by inhibiting eIF4E from recruiting 40S ribosomal subunits during translation [34–36]. Upon phosphorylation, 4EBP1 dissociates from eIF4E allowing for active cap-dependent translation [37–40].

Interestingly, many human cancers [41, 42], and particularly breast cancers with the 8p11-p12 amplicon overexpress 4EBP1 [43] [44]. Since 4EBP1 inhibits translation, it is expected that overexpression of 4EBP1 would act as a tumor suppressor. However, overexpression of 4EBP1 results in high levels of phosphorylated 4EBP1 which may contribute to breast cancer development [43, 45] [44–47]. Indeed, proteins that can regulate 4EBP1 phosphorylation, like Casein kinase 1^ε^ [48, 49], Glycogen synthase kinase (GSK)-3β [50], G1 To S phase transition 2 (eRF3b) [51, 52], Mammalian target of rapamycin complex 1 (mTORC1) [39, 40, 53–60], Polo like kinase 1 (PLK1) [61–63], Family with sequence similarity 129 member A (Niban) [64], PI3-kinase isoforms [65, 66], Cyclin-dependent kinase 1 (CDK1) [59, 67–70], ATM serine/threonine kinase (ATM) [71, 72], Mitogen activated protein kinase (MAPK) [73, 74], Protein kinase B (AKT) [75], and others [68, 74, 76] have been suggested as therapeutic targets for cancer. Given the relationship between expression of 4EBP1 in the 8p11-p12 amplicon and hyperactivation of mTORC1 observed in endocrine resistant breast cancers, PI3K/AKT/mTORC1 targeted therapies have been suggested for 4EBP1 expressing breast cancers [46, 77–81]. Furthermore, genes within the amplicon as well as mTORC1, which phosphorylates 4EBP1, have been shown to activate ER, potentially contributing to the ability of amplicon bearing breast cancer cells to circumvent endocrine therapy.

Consequently, we set out to evaluate the effect of 4EBP1 targeting in ER+, 8p11-p12 expressing breast cancer cells as well as other breast cancer cell lines, and non-tumorigenic but immortalized human mammary epithelial cells. We first found that 4EBP1 is an essential gene in the SUM-44 cells based on results of a genome-scale shRNA screen, and then found that 4EBP1 targeting reduced proliferation of not only amplicon bearing cells (SUM-44, Cama-1, SUM-52) but also non-amplicon ER+ breast cancer cells as well (MCF7, T47D). This effect was also seen in ER-negative (ER-) 8p11-p12 cells (SUM-52) as well as non-amplicon bearing cells (SUM-229, SUM-149), but to a lesser extent. There was no effect of 4EBP1 targeting on the proliferation of immortalized but non-tumorigenic mammary epithelial cells (MCF10A, H16N2). Consistent with our findings, Kaplan-Meier analysis shows that high levels of 4EBP1 correlates with worsened prognosis in ER+ cohorts (ER+, ER+ Luminal A, and ER+ Luminal B) as well as cohorts that received chemotherapy, tamoxifen, or endocrine therapy. Taken together, our findings suggest that 4EBP1 plays an important role in in breast cancer and may be particularly important in breast cancers with the 8p11-p12 amplicon regardless of ER status.

## Methods

### Antibodies and inhibitors

The mTOR inhibitor, Everolimus (RAD001), was purchased from Selleckchem (S1120, A112024). The antibodies against 4EBP1 (9644), phospho-4EBP1 Ser65 (9451), phospho-4EBP1 Thr37/46 (2855), phospho-4EBP1 Thr70 (9455) were purchased from Cell Signaling. Antibody against β-actin (A5441) was purchased from Sigma-Aldrich. The CyclinD1 (2978) and p27 Kip1 (3686) antibodies were purchased from Cell Signaling. The ERα antibody (sc-543) was purchased from Santa Cruz Biotechnology.

### Cell culture

The SUM-44 (ER+), Cama-1 (ER+), and SUM-52 (ER-) cell lines represent luminal breast cancer and have the 8p11-p12 genomic locus amplified. T47D (ER+), HCC1500 (ER+), and MCF7 (ER+) cells are also luminal but 8p11-p12 is not amplified. SUM-229 and SUM-149 are triple-negative breast cancer cell lines. Normal breast epithelial are represented by immortalized but non-tumorigenic MCF10A and H16N2 cell lines. All cell lines were maintained at 37 °C with 10% CO_2_. SUM cell lines and culture requirements for maintenance with Hams F12 cell culture medium (Hyclone SH30026FS, Thermo Fisher Scientific) with supplementation have been previously described [82–84] (please refer to the SLKBase (https://sumlineknowledgebase.com/) for additional information about these cell lines). The Cama-1 cell line (obtained from ATCC) and MCF7 cell line (obtained from the Michigan Cancer Foundation) were grown in Dulbecco’s Modified Eagle’s (DMEM) medium (obtained from Thermo Fisher Scientific) containing 10% Fetal Bovine Serum (FBS) purchased from Gemini Bioproducts (900–108) or Atlanta Biologicals (S11050). The T47D cell line (obtained from ATCC) was grown in Roswell Park Memorial Institute (RPMI) medium (Thermo Fisher Scientific) containing 10% FBS. The MCF10A cells were obtained from Dr. Herb Soule at the Michigan Cancer Foundation [85] and were maintained in serum-free Hams F12 supplemented with Bovine serum albumin (BSA) (126,579, Millipore), 5 μg/mL Insulin (700-112P, Gemini Bioproducts), 1 μg/mL Hydrocortisone (H-4001, Sigma-Aldrich), and 10 ng/mL Epidermal Growth Factor (E9644, Sigma-Aldrich) (SFIHE medium). H16N2 cells [86, 87] (immortalized by human papillomavirus (HPV) E6 and E7 oncoproteins) were also grown in SFIHE medium. When trypsinizing cells grown in serum-free medium, 2% FBS was added for the first 24 h. The SUM cell lines were developed in the author’s laboratory and are routinely validated for identity by STR profiling. The remaining cell lines were obtained from ATCC and were used immediately upon arrival. All cell lines are routinely tested for mycoplasma.

### Generation of *EIF4EBP1* knockdown cells

Lentivirus was produced in 293FT cells which were transfected in Opti-MEM with Lipofectamine 2000, pLKO.1-puro gene-targeting plasmid, and Mission packaging mix (Sigma-Aldrich) under optimal conditions. Collected virus was filtered through a 0.2 um filter before storage at − 80 °C. Efficient viral titer production was confirmed by a Lenti-X p24 Rapid Titer Kit (Takara) and 4EBP1 western blot. All BSL-2 safety protocols were performed during production, storage, and continued use. Optimization was performed with listed (Table 1) 4EBP1-targeting plasmids wherein TRCN0000040206 (4EBP sh_1) and TRCN0000298904 (4EBP_sh_2) produced efficient knockdown and were used for subsequent studies. These were obtained from the shRNA Technology Shared Resource (Hollings Cancer Center, the Medical University of South Carolina).

**Table 1:** ᅟ.

Plasmid	Genotype	Region	Sequence
shLACZ	pLKO.1-puro::*LACZ*	n/a	CGCTAAATACTGGCAGGCGTT
Sh4EBP1 #1	pLKO.1-puro::*EIF4EBP1* TRCN0000040206	CDS	CCGGCGGTGAAGAGTCACAGT TTGACTCGAGTCAAACTGTGA CTCTTCACCGTTTTTG
Sh4EBP1 #2	pLKO.1-puro::*EIF4EBP1* TRCN0000040203	3UTR	CCGGGCCAGGCCTTATGAAA GTGATCTCGAGATCACTTTC ATAAGGCCTGGCTTTTTG
Sh4EBP1 #3	pLKO.1-puro::*EIF4EBP1* TRCN0000310343	3UTR	CCGGGCCAGGCCTTATGAAA GTGATCTCGAGATCACTTTC ATAAGGCCTGGCTTTTTG
Sh4EBP1 **#**4	pLKO.1-puro::*EIF4EBP1* TRCN0000298904	CDS	CCGGCGGTGAAGAGTCACAG TTTGACTCGAGTCAAACTGT GACTCTTCACCGTTTTTG

Cells were reverse transfected with lentivirus, with appropriate growth medium, and polybrene. Virus was removed 24 h later and cells were fed with media. Cells began selection with appropriate concentration of antibiotics 48 h following transfection. Antibiotic concentration at 2 μg/ml Puromycin (invivoGen) was sufficient to ensure selection. The SUM-44 cell line requires 3 μg/ml Puromycin selection. Control cells without the addition of lentivirus were plated alongside lentivirus infected cells to ensure the appropriate concentration of antibiotic was used. Cells were continuously maintained in the resistance marker. All further parameters were tested after four days of selection in Puromycin.

### Cell proliferation

Cells were plated in 12-well plates at [1E5 cells/well], washed with 1X Phosphate Buffered Saline (PBS), then 0.5 mL HEPES/MgCl_2_ buffer (Isoton) was added to each dish and agitated for 5 min. Cell swelling was confirmed and 50 uL ZAP (Bretol Solution) was added and incubated for 10 min with agitation. Cells were visualized to confirm bursting and nuclei release, 10 mL NaCl-Formalin Solution was added to prevent deterioration, and read using a Coulter Acuvette. The Coulter Counter was set to count nuclei between 4 and 8 um diameter through a 100 um aperture. Each sample was counted twice and then averaged. The counts were multiplied by 20 to obtain the total number of nuclei, and background counts with NaCl-Formalin were performed with analysis.

### Statistical analysis

Growth results were analyzed using a two-way ANOVA model with an interaction effect between day and condition. Each cell line was analyzed individually and all analyses were done on the log scale. Differences between conditions were exponentiated to obtain fold change estimates. Significance testing was completed using Tukey’s honestly significant difference method to maintain a family-wise alpha of 0.05 within each cell line.

### Immunoblotting

Cells were continuously maintained on ice and harvested using Radioimmunoprecipitation assay (RIPA) buffer (Sigma-Aldrich) supplemented with a protease inhibitor cocktail (Millipore, #539131) and PhosSTOP (Roche). Bradford Protein Assay was used to fit samples to a standard curve and determine protein concentrations prior to SDS-PAGE. After transfer, PVDF membrane was blocked 1 h with 5% skim milk in 1X TBST at room temperature and incubated overnight with antibody per the manufacturer’s instructions. The membrane was visualized with SuperSignal West Pico Chemiluminescent Substrate (Thermo Fisher Scientific). The membrane was developed using the Li-COR Odyssey Fc.

### Flow cytometry of live cells

Cells were trypsinized, counted, and analyzed at [1E6 cells/mL]. Vybrant DyeCycle Orange (V35005, Thermo Scientific) was used according to the manufacturer’s protocol for live cell-cycle analysis. Conditions were optimized to a final stain concentration of 5 μM in 1X PBS in all cell lines tested. Cells were promptly analyzed using a BSL2 FACSAria Cell Sorter. Verity ModFit LT 4.1 was used to analyze and visualize the generated data.

### KM plotter database analysis

The KM plotter for breast cancer (http://kmplot.com) [88] was used on all releases available from the database accessed spring 2018. Restricted analyses of different populations are indicated and altered the number of breast cancer patients with available survival data as shown by the number at risk. The determined and represented prognostic values by relapse free survival (RFS) of *EIF4EBP1* in all analyses were more than 500 samples, indicating highly reliable analysis using all parameters presented. The JetSet best probe set for *EIF4EBP1* (probe ID: 221539_at) was used for all analyses. Patients were divided into a high and low expression group by median mRNA expression values, all possible cutoff values between the lower and upper quartiles were computed and the best performing threshold was determined by using auto select the best cutoff. RFS was plotted using suggested quality controls. This excluded biased arrays, removed redundant samples, and checked proportional hazards assumptions. The cutoff values, probe expression range, false discovery rate (FDR), and *p*-value were extracted from the KM plotter webpage and each analysis is represented.

### cBioPortal database analysis

**Fig. 1:**
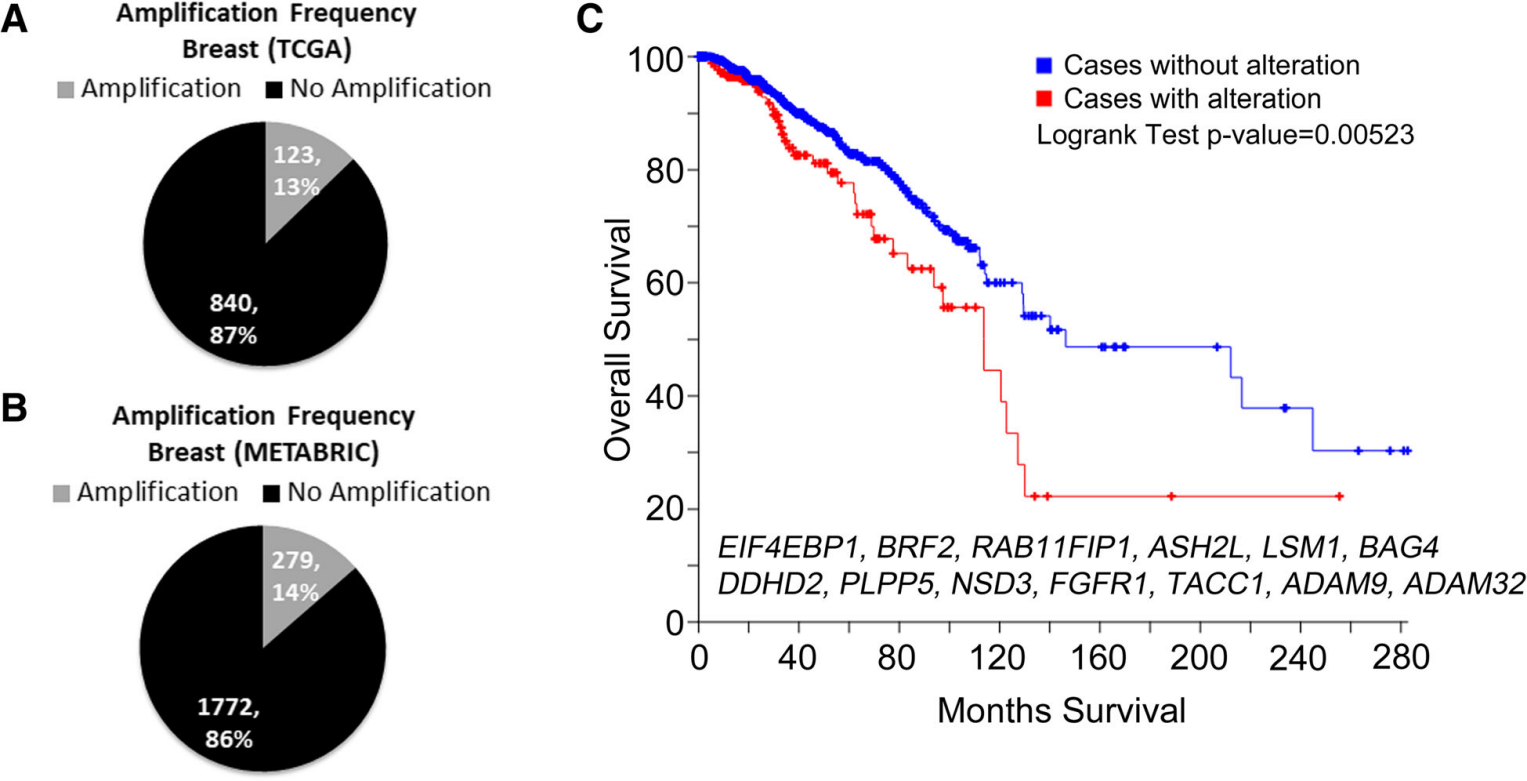
*EIF4EBP1* is amplified in human breast cancer and correlates with reduced overall survival. (**a**) Amplification data from The Cancer Genome Atlas (TCGA) and (**b**) the Molecular Taxonomy of Breast Cancer International Consortium (METABRIC). (**c**) Expression analysis using TCGA provisional data shows that high expression of A2 genes: *BRF2, RAB11FIP1, EIF4EBP1, ASH2L, LSM1, BAG4, DDHD2, PLPP5, NSD3, FGFR1, TACC1, ADAM9,* and *ADAM32* correlates with reduced overall survival

The cBioPortal(http://www.cbioportal.org/) [89, 90] was used to generate the overall survival curve (shown in Fig. 1b) for breast tumors with and without A2 8p11-p12 region alterations using The Cancer Genome Atlas (TCGA) provisional data. The Amplification frequency of 4EBP1 in the TCGA or the Molecular Taxonomy of Breast Cancer International Consortium (METABRIC) breast data cohorts were also determined using the cBioPortal. Data was accessed spring 2018.

## Results

### Frequency and prognostic significance of 4EBP1 amplification in breast cancer

*EIF4EBP1*, the gene that encodes the 4EBP1 protein, resides within the 8p11-p12 genomic locus. It is frequently amplified in endocrine resistant luminal breast cancers, rarely coincides with *PIK3CA* mutations, and is associated with poor prognosis. The frequency of *EIF4EBP1* amplification across all breast cancer subtypes is approximately 13% according to data from The Cancer Genome Atlas (TCGA) and 14% according to data from the Molecular Taxonomy of Breast Cancer International Consortium (METABRIC) [91] (Fig. 1a & b). Furthermore, we found that expression analysis of the TCGA provisional data shows that high expression of the genes in the A2 region of the 8p11-p12 amplicon, which includes *EIF4EBP1, BRF2, RAB11FIP1, ASH2L, LSM1, BAG4, DDHD2, PLPP5, NSD3, FGFR1, TACC1, ADAM9,* and *ADAM32,* correlates with reduced overall survival (Fig. 1c).

### 4EBP1 is highly expressed and phosphorylated in 8p11-p12 breast cancer cells

**Fig. 2:**
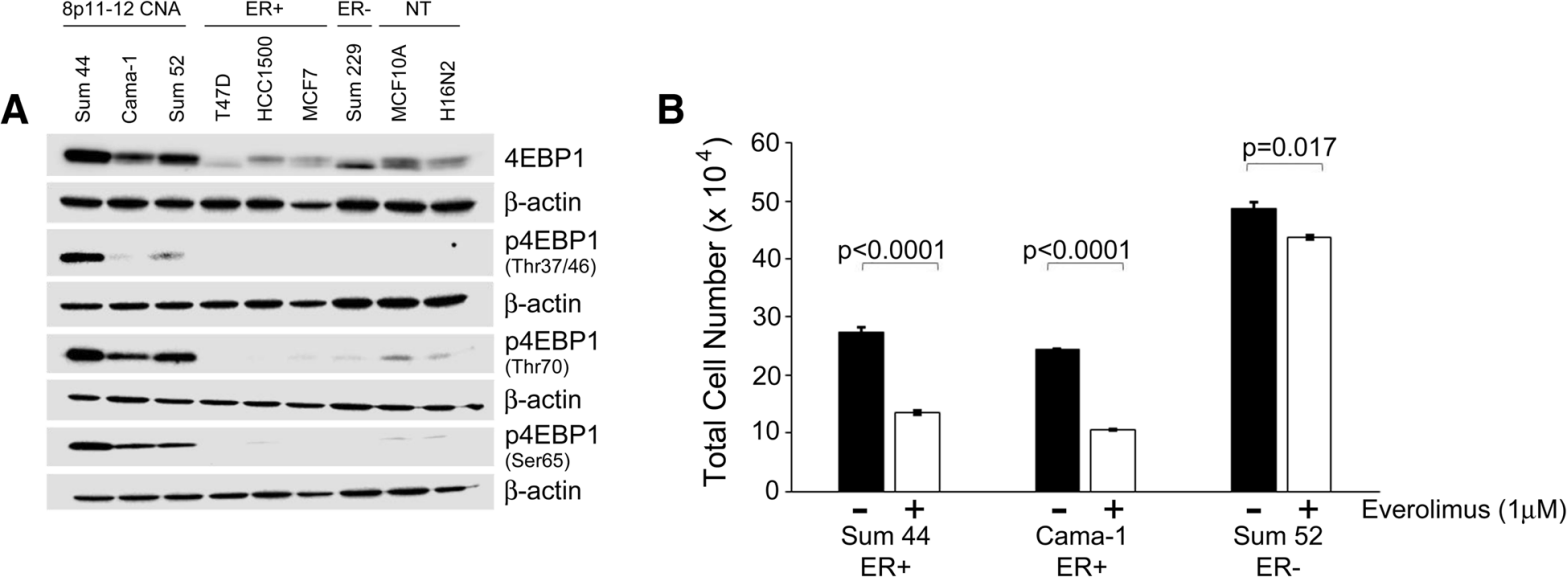
4EBP1 is highly expressed and phosphorylated in 8p11-p12 breast cancer cells. (**a**) Western blot of 4EBP1 and phospho-4EBP1 on residues Thr 37/37, Thr 70, and Ser 65 in SUM-44 (ER+), Cama-1 (ER+), and SUM-52 (ER-) cells with amplification of the 8p11-p12 genomic locus (8p11-12 CNA) as well as T47D (ER+), HCC1500 (ER+), MCF7 (ER+), and SUM-229 (ER-) cells without amplification of the 8p11-p12 genomic locus. Immortalized but non-tumorigenic breast epithelial cells are represented by MCF10A and H16N2 cells. (**b**) Cell proliferation was assessed in SUM-44, Cama-1, and SUM-52 cells in the presence or absence of 1 μM everolimus treatment for 72 h. Error bars represent standard deviation among replicates and *p* values are for the difference in cell proliferation in control versus treated cells. The *p*-value for the difference between the effect in SUM-52 cells and the other cell lines is <0.0001

To investigate the significance of 4EBP1 overexpression in breast cancer, we employed a set of human breast cancer cell lines representing ER+ and ER- samples, including SUM-44, SUM-52, and Cama-1 (ER+, amplicon bearing), MCF-7, T47D, and HCC1500 (ER+, non-amplicon bearing), and SUM-229, as well as two non-tumorigenic but immortalized mammary epithelial cell lines, MCF10A, and H16N2 cells. As expected, SUM-44, Cama-1, and SUM-52 expressed high levels of 4EBP1 due to the amplification of the *EIF4EBP1* gene, whereas MCF10A and H16N2 did not express any more or less 4EBP1 protein than the T47D, HCC1500, MCF7, or SUM-229 cell lines (Fig. 2a). High levels of phosphorylated 4EBP1 were also readily detected in the SUM-44, Cama-1 and SUM-52 cells compared to the other cell lines tested (Fig. 2a).

4EBP1 is thought to be phosphorylated by mTORC1 in a hierarchical fashion [39, 40]. Our findings that 4EBP1 expression and phosphorylation levels are high on multiple residues in SUM-44, Cama-1, and SUM-52 cells, as well as our observation of high levels of phospho-S6 (not shown) suggest active mTORC1 signaling in these 8p11-p12 models. Therefore, we tested the effect of mTOR pathway inhibition on cell proliferation of the 8p11-p12 cell lines. Cells were plated in equal number and on day 1 after plating, cells were exposed to 1 uM of the inhibitor everolimus (Affinitor). To assess proliferation, the total number of cells was quantitated for each group at day 1, prior to treatment, and on day 4, 72 h after exposure to everolimus. Treatment with everolimus significantly reduced the proliferation of all three cell lines, however the fold-change observed for the SUM-44 cells and the Cama-1 cells (0.49 and 0.43 respectively) were significantly greater than the fold change observed in the SUM-52 cells (0.9). The difference in response to everolimus between the SUM-52 cells and the other two cell lines was significant with a *p*-value of < 0.0001. (Fig. 2b). This result suggests that ER expression plays a role in the responsiveness of breast cancer cells to everolimus.

### 4EBP1 is essential to breast cancer cell lines

Our laboratory recently completed a genomic scale shRNA screen for the entire panel of SUM breast cancer cell lines, and some of the results from these screens have been reported elsewhere [92] and can be found at The SUM Breast Cancer Cell Line Knowledge Base (SLKBase) (https://sumlineknowledgebase.com/) [93]. Interestingly, despite the fact that the SUM-44 cells have been shown to overexpress several genes from the 8p11-p12 amplicon that can behave as transforming oncogenes in vitro, EIF4EBP1 was the strongest hit among all 8p11 amplified genes in this RNA interference screen. The DepMap [111, 112, 113] crispr (Avana) gene essentiality screens also predict 4EBP1 as a driver of cancer cell lines including all of the breast cancer cell line models currently represented within the portal (https://depmap.org/portal/) [94] Therefore, we performed experiments to validate the importance of 4EBP1 knockdown in SUM-44 cells and extended that to other breast cancer cell lines. To gain a broader understanding of 4EBP1 in different settings, we performed experiments to assess the effect of 4EBP1 knock-down on proliferation of cell lines that represent different subtypes of breast cancer.

**Fig. 3:**
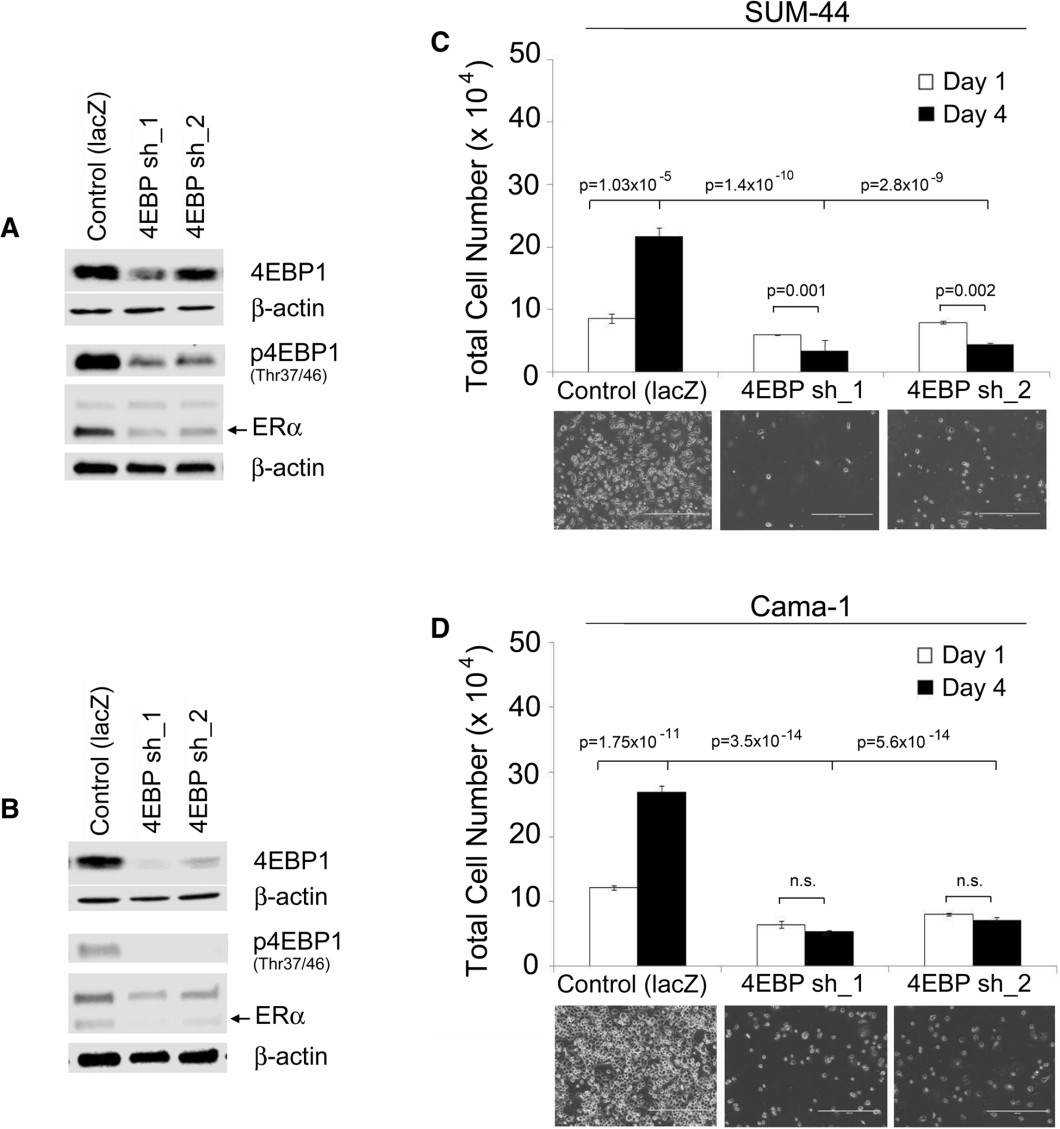
4EBP1 knockdown inhibits proliferation of ER+ 8p11-p12 breast cancer cells. (**a**) Western blot of 4EBP1, phospho-4EBP1 on residues Thr 37/46, and ERα in SUM-44 cells engineered with either control shRNA to *lacZ* or two individual shRNAs to *EIF4EBP1* (4EBP sh_1 or sh_2). (**b**) Western blot of 4EBP1, phospho-4EBP1 on residues Thr 37/46, and ERα in Cama-1 cells engineered with either control shRNA to *lacZ* or two individual shRNAs to *EIF4EBP1* (4EBP sh_1 or sh_2). (**c**) Cell proliferation was assessed in SUM-44 and (**d**) Cama-1 control and *EIF4EBP1* knockdown cells on day 1 and day 4 in culture following selection in puromycin containing media. Error bars represent standard deviation among replicates and *p*-values represent the statistical comparison between each corresponding group

To determine the effect of directly targeting 4EBP1 in breast cancer cells, we first tested the two ER+ 8p11-p12 cell lines, SUM-44 and Cama-1, and used lentiviral vectors for two different shRNAs against EIF4EBP1. shRNA targeting *lacZ* was used as a control. Fig. 3 shows that both shRNAs were effective at reducing levels of 4EBP1 protein, and there was a concomitant decrease in the levels of phosphorylated 4EBP1 (Fig. 3 a & b). We then measured proliferation of cells expressing *EIF4EBP1* shRNA compared to control cells. Cells were evaluated by counting the number of nuclei at day 1 and day 4 after plating. The data shown in Fig. 3 c and d show that there as a significant increase in cell number in the LacZ control cells over the 4-day culture period, there was little or no proliferation in the sh4EBP1 groups in either cell line. Indeed, there was a significant reduction in cell number over the 4 day period in the SUM-44 cells (fold change = 0.5, *p* < 0.001, 0.002), whereas in the Cama-1 cells, there was a smaller (approximately 0.8 fold) but still significant difference in cell number over the same period (*p* < 0.002, and 0.07). The largest and most statistically significant difference was detected in the day 4 cell counts between control LacZ cells and the sh4EBP1 cells in both cell lines, with fold-differences of approximately 4 and 6-fold, and *p*-values ranging from 10^− 9^ to 10^–14^ The full ANOVA analysis of the data for all groups and all time points are shown in Additional file 3: Table S1.

Prior studies from our lab and others have demonstrated the effects of genes associated with the 8p11-p12 amplicon on ERα expression [1, 28–31, 100]. Therefore, we next evaluated ERα expression in the SUM-44 and Cama-1 *EIF4EBP1* knockdown cell lines and found that ERα levels were reduced (Fig. 3 a & b) compared to control cells expressing *lacZ* shRNA. These findings show that reducing 4EBP1 levels impairs proliferation of the ER+ 8p11-p12 breast cancer cell models and results in downregulation of ERα.

**Fig. 4:**
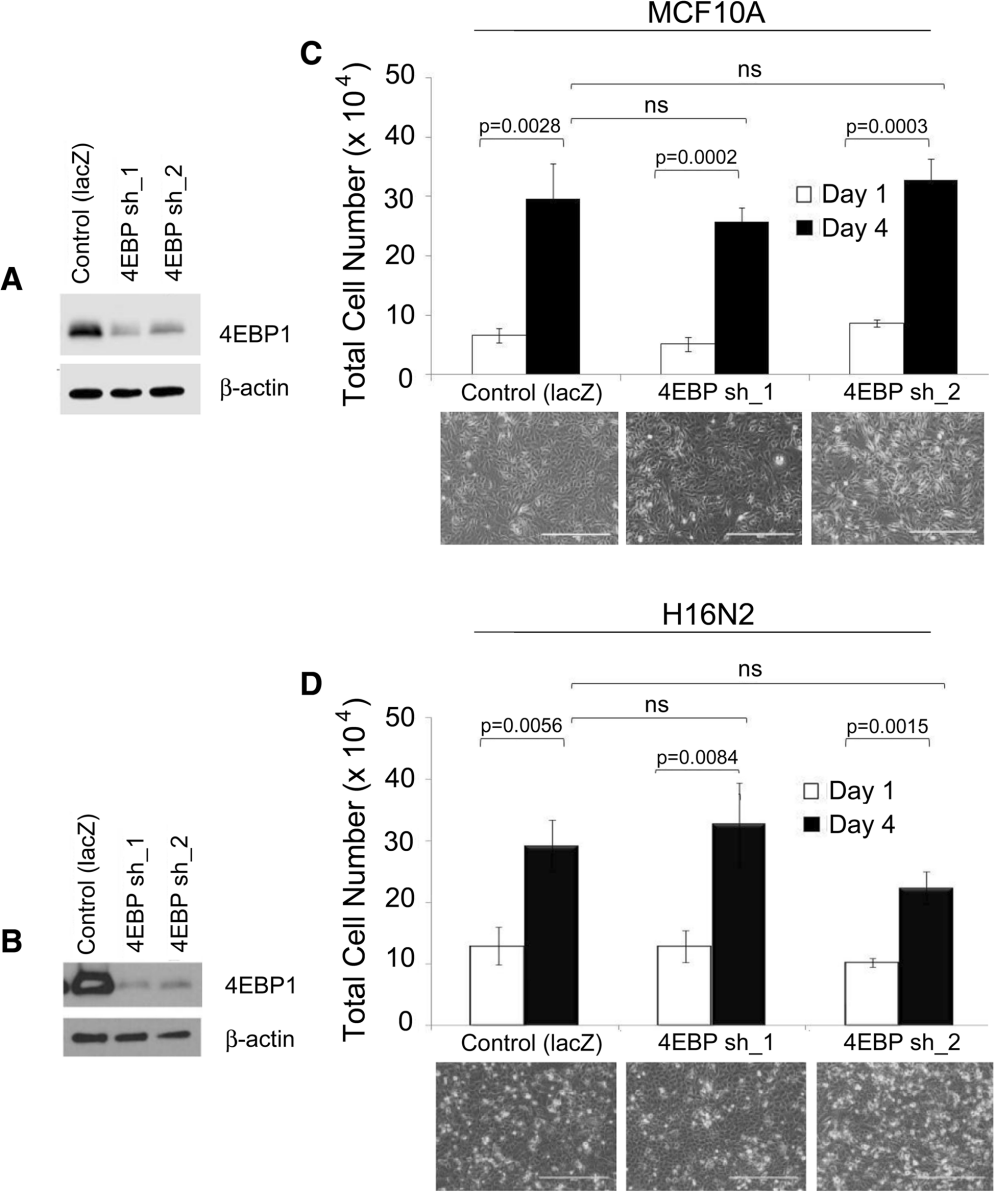
4EBP1 knockdown does not affect proliferation of MCF10A and H16N2 non-transformed breast epithelial cells. (**a**) Western blot of 4EBP1 in MCF10A cells and (**b**) H16N2 cells engineered with either control shRNA to *lacZ* or two individual shRNAs to *EIF4EBP1* (4EBP sh_1 or sh_2). (**c**) Cell proliferation was assessed in MCF10A and (**d**) H16N2 control and *EIF4EBP1* knockdown cells on day 1 and day 4 in culture following selection in puromycin-containing medium. Error bars represent standard deviation among replicates and p-values represent significance between each corresponding group

We next wanted to evaluate the potential effects of 4EBP1 targeting in non-tumorigenic human breast epithelial cells. 4EBP1 was knocked down in MCF10A (Fig. 4a) and H16N2 cells (Fig. 4b). Cell proliferation was then measured by counting the total number of cell nuclei present at day 1 and day 4 after plating. All populations increased in number significantly over four days and no statistically significant differences were observed between control and *EIF4EBP1* knockdown in MCF10A cells (Fig. 4c) or H16N2 cells (Fig. 4d). These results indicate that downregulation of 4EBP1 in non-tumorigenic breast epithelial cell lines, at least to the same levels as was achieved in the breast cancer cell lines does not affect the proliferative capacity of these cells.

### Downregulation of 4EBP1 in ER+ 8p11-p12 breast cancer cells causes cell cycle arrest

**Fig. 5:**
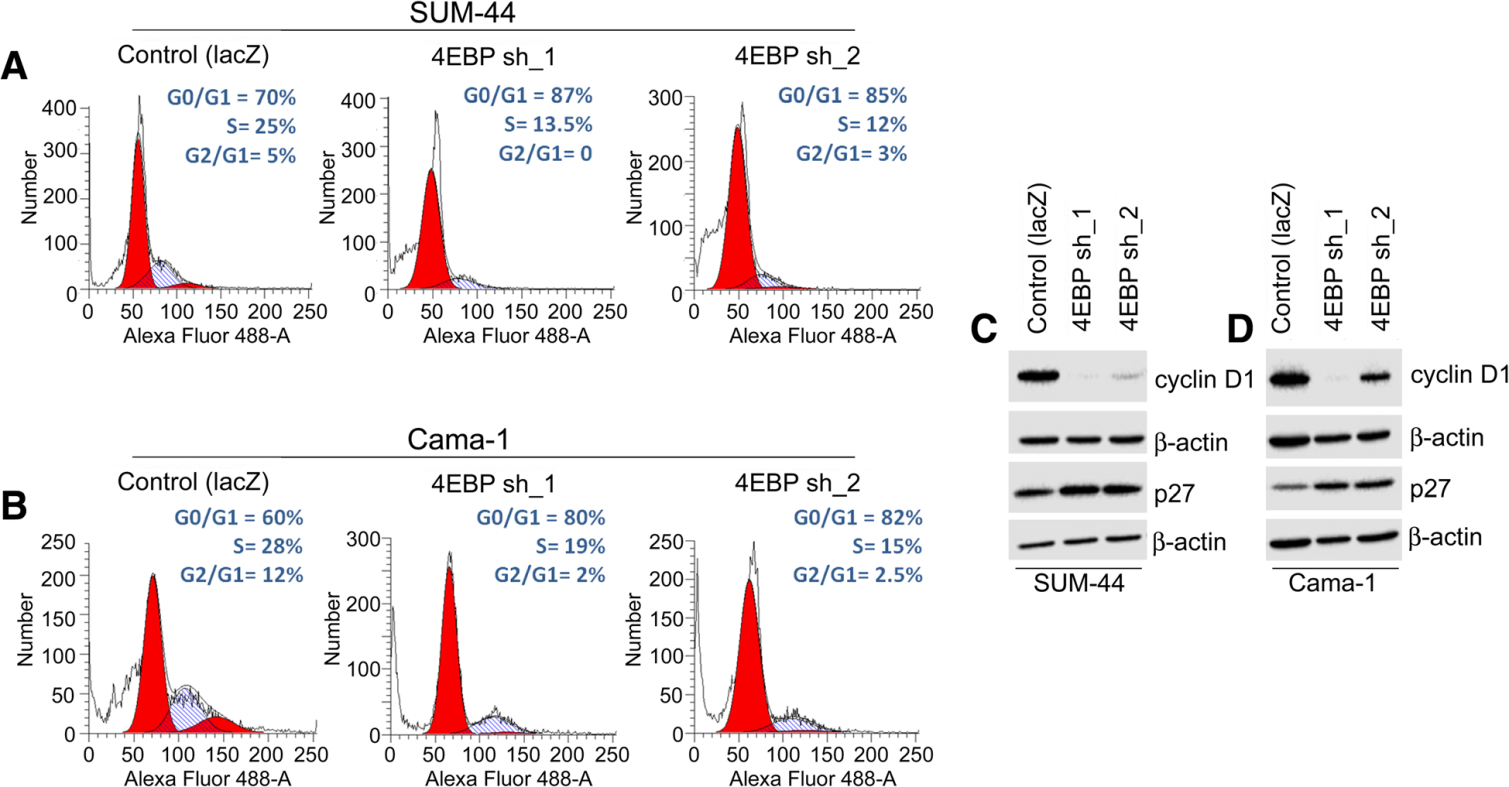
4EBP1 knockdown leads to G0/G1 arrest in ER+ 8p11-p12 breast cancer cells. (**a**) Cell cycle analysis of SUM-44 and (**b**) Cama-1 cells shows that 4EBP1 knockdown results in an accumulation of cells in G0/G1 with an associated decrease in cells in S-phase. (**c**) Western blot of cyclin D1 and p27 in SUM-44 and (**d**) Cama-1 cells engineered with either control shRNA to *lacZ* or two individual shRNAs to *EIF4EBP1* (4EBP sh_1 or sh_2)

Previous studies suggest that 4EBP1 regulates cell cycle progression [59, 61, 68, 101–104]. To better understand the cellular effects of 4EBP1 knockdown, SUM-44 and Cama-1 cells were assessed by flow cytometry to evaluate cell cycle progression. An increase in the number of cells in G1 cell-cycle in both SUM-44 (Fig. 5a) and Cama-1 cells (Fig. 5 b) was observed with *EIF4EBP1* knockdown when compared to control cells. These results show that knockdown of 4EBP1 promotes G1 cell cycle arrest.

To study the cell cycle arrest induced by 4EBP1 knock-down further, we evaluated the protein expression levels of key cell cycle regulators. We found that Cyclin D1 protein levels were decreased in SUM-44 and Cama-1 cells following *EIF4EBP1* knockdown (Fig. 5c & d). Additionally, we observed a slight increase in p27 levels in the *EIF4EBP1* knockdown cells compared to control cells (Fig. 5c & d). The alterations of Cyclin D1 and p27 expression that we found are consistent with the cell cycle arrest phenotype that we observed in 4EBP1 knockdown cells.

### 4EBP1 knockdown inhibits proliferation of ER- 8p11-p12 amplified breast cancer cells

**Fig. 6:**
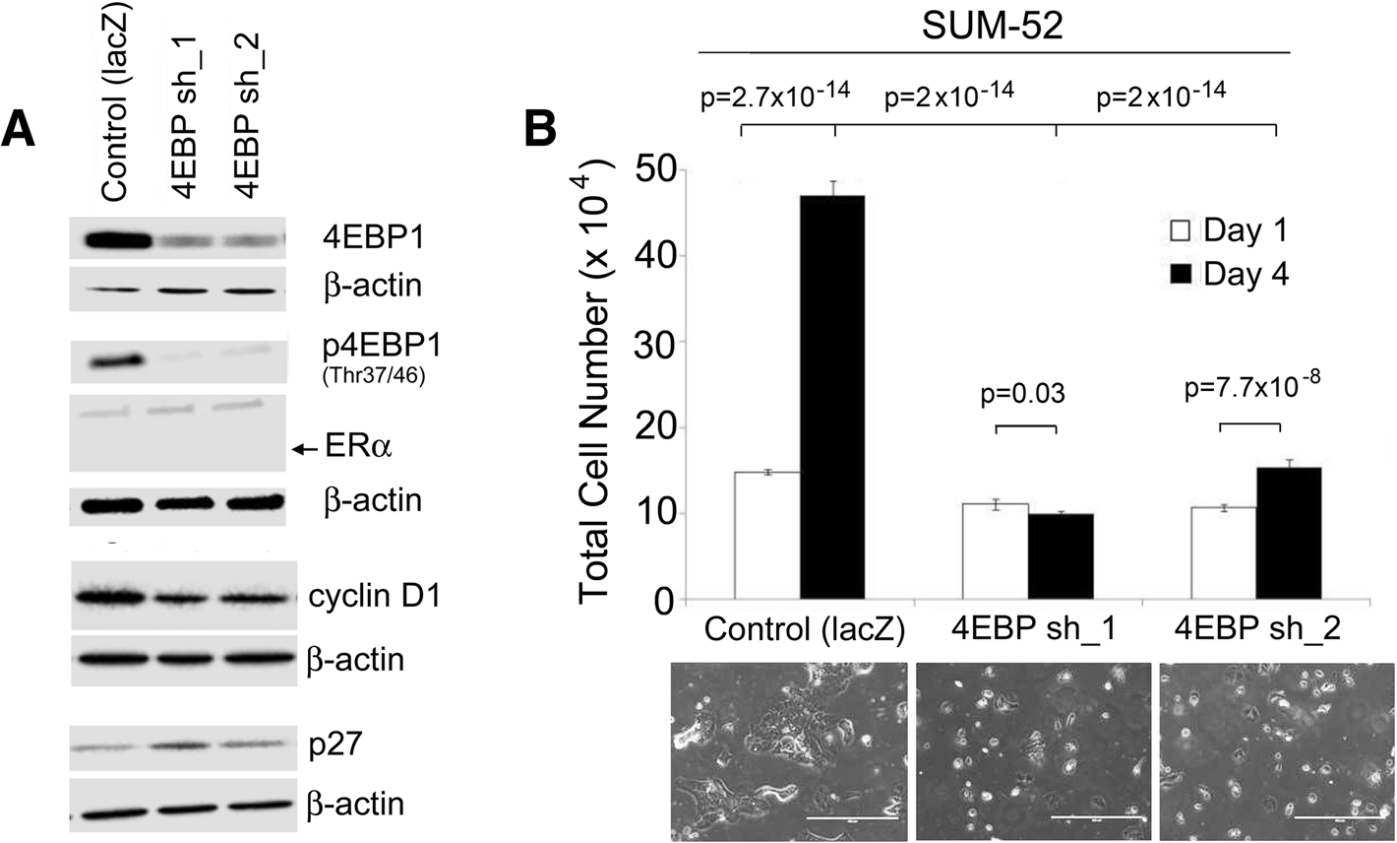
4EBP1 knockdown inhibits proliferation of ER- 8p11-p12 breast cancer cells. (**a**) Western blot of 4EBP1, phospho-4EBP1 on residues Thr 37/46, ERα, cyclin D1, and p27 in SUM-52 cells engineered with either control shRNA to *lacZ* or two individual shRNAs to *EIF4EBP1* (4EBP sh_1 or sh_2). (**b**) Cell proliferation was assessed in SUM-52 control and *EIF4EBP1* knockdown cells on day 1 and day 4 in culture after selection in puromycin-containing medium. Error bars represent standard deviation among replicates and significance is the comparison between each corresponding group

Because we saw only a small effect of everolimus on the proliferation of the ER- 8p11-p12 SUM-52 breast cancer cell line, we also wanted to test the effect of *EIF4EBP1* knockdown on these cells. Using the same two shRNAs targeted to *EIF4EBP1* as we used on the previous cell lines, we knocked down 4EBP1 mRNA in the SUM-52 cells and likewise, saw a reduction in 4EBP1 protein levels (Fig. 6a). *EIF4EBP1* knockdown in SUM-52 cells resulted in a dramatic reduction in proliferation of SUM-52 cells, similar to what we observed with the two ER+ cell lines. In LacZ control cells, there was a highly significant increase in cell number between days 1 and 4, whereas in the sh4EBP1 cells, there was a slight reduction in cell number in the sh1 group and a slight increase in cell number is the sh2 group. These differences most likely reflect different levels of knockdown achieved with the two vectors. Of greatest importance is the three to four-fold difference in the number of cells per dish at the 4 day time point between the shLacZ and sh4EBP1 groups again with *p*-values on the order of 10^− 14^. (Fig. 6b). We also probed these control and knockdown cells for Cyclin D1 and p27 expression. We saw a similar effect on these two proteins as in the SUM-44 and Cama-1 cells where Cyclin D1 levels were decreased and p27 levels were increased (Fig. 6a). We also evaluated the effect of 4EBP1 knockdown on the non-amplicon bearing models, MCF7 (ER+) (Additional file 1: Figure S1 a), T47D (ER+) (Additional file 1: Figure S1, b), SUM-229 (ER-) (Additional file 2: Figure S2 a), and SUM-149 (ER-) (Additional file 2: Figure S2 b). These experiments showed that knockdown of 4EBP1 in MCF7 and T47D also significantly inhibited proliferation (Additional file 1: Figure S1 c & d). By contrast, 4EBP1 knock-down in the triple negative SUM-149 and SUM-229 cells was less effective at reducing proliferation of these cells (Additional file 2: Figure S2 c & d).

### *EIF4EBP1* expression levels correlate with reduced relapse free survival in human breast cancer

**Fig. 7:**
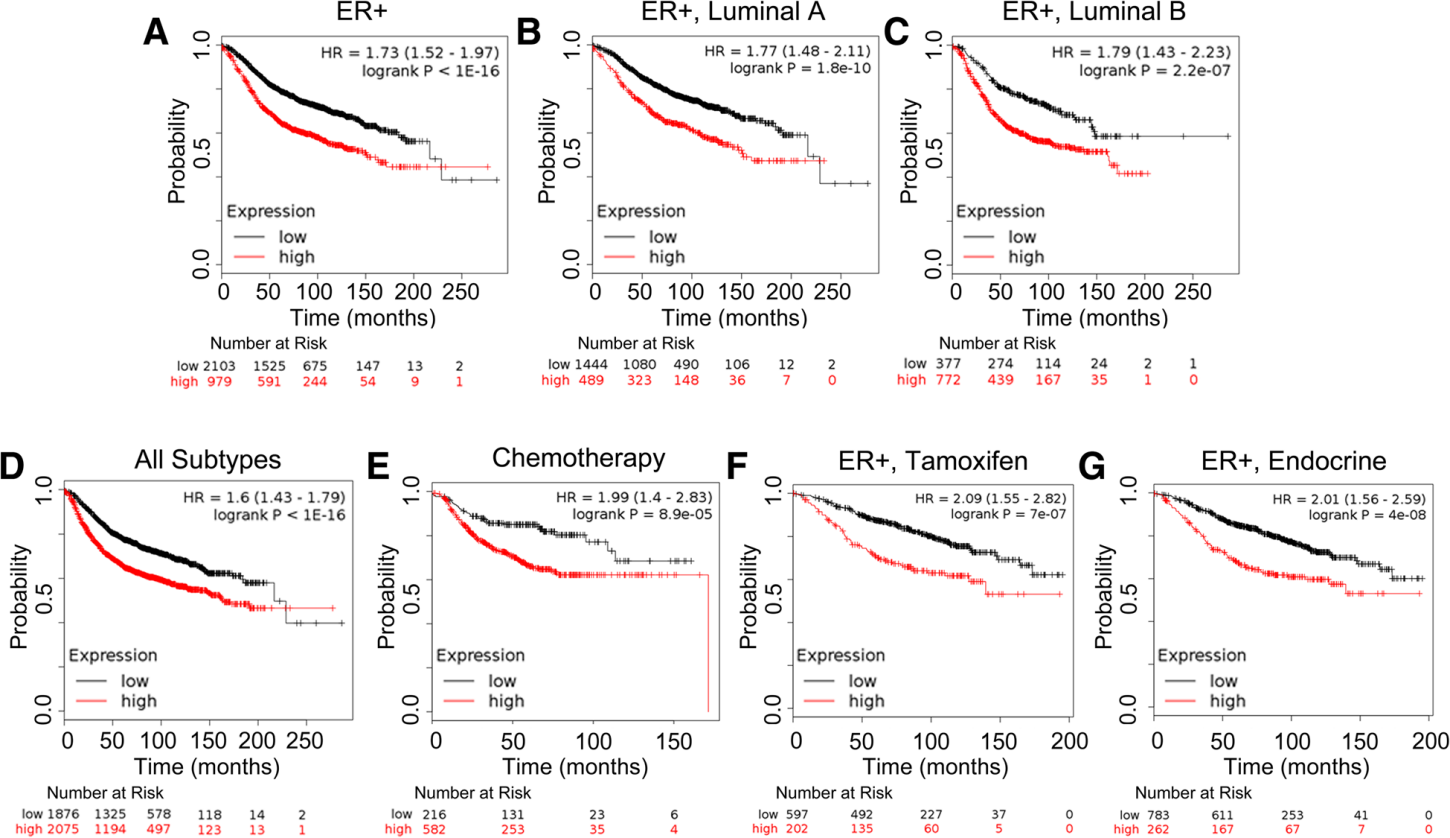
Kaplan-Meier analysis of breast cancer outcomes in patients with and without overexpression of 4EBP1. KM plotter analysis of *EIF4EBP1* (probe ID: 221539_at) gene expression and overall survival in (**a**) ER+ populations (**b**) separated by luminal A (**c**) luminal B subtypes (**d**) all subtypes (no parameters selected) (**e**) post treatment with chemotherapy (**f**) tamoxifen or (**g**) endocrine therapy

To determine the overall impact of *EIF4EBP1* on survival and to assess whether treatment affects the outcomes, we used the online Kaplan-Meier plotter database tool (kmplot.com) to assess the relationship between *EIF4EBP1* gene expression and relapse free survival. This tool uses gene expression data from Gene Expression Omnibus (GEO), the European Genome-phenome Archive (EGA), and The Cancer Genome Atlas (TCGA) [88].The JetSet probe set for *EIF4EBP1* (probe ID: 221539_at) was used for all analyses. We found that high *EIF4EBP1* gene expression significantly correlated with reduced relapse free survival not only in ER+ populations (Fig. 7a), including when separated by luminal A (Fig. 7b) and luminal B (Fig. 7c) subtypes, but also across all subtypes (Fig. 7d). Furthermore, this was also true post treatment with chemotherapy (Fig. 7e) and following either tamoxifen (Fig. 7f) or other endocrine therapy (Fig. 7g). Altogether, these analyses point to a role of 4EBP1 overexpression in breast cancer development and response to therapy.

## Discussion

We and others have determined that a number of oncogenes reside within the 8p11-p12 region and are amplified in human breast cancer. Genes found within this region such as *WHSC1L1* [11], *DDHD2* [11], *LSM1* [10, 11, 18], *BAG4* [10, 11], and *KAT6A* [16, 28] have all been shown to have transforming properties in vitro*.* Of significance, the 8p11-p12 amplicon is implicated in endocrine resistance [1]. Consistent with this implication, NSD3 (aka *WHSC1L1*) was shown to drive high levels of ER expression, and to enhance proliferation in an estrogen independent manner [29]. Reminiscent of this finding, hyperactive mTOR is often observed in endocrine resistant cells and can activate ERα [95–99]. Interestingly, the *EIF4EBP1* gene which encodes the mTOR effector protein 4EBP1 is located on the short arm of chromosome 8 within the 8p11 region of the amplicon. It is highly overexpressed but rarely mutated in breast cancer, regardless of amplification, and has been suggested to be an essential driving gene in many cancer cell lines in vitro which we [93]and others have witnessed [94]using genome-wide gene essentiality screens. Consequently, our study initially aimed to determine whether 4EBP1 overexpression influences proliferation in ER+ 8p11-p12 amplicon positive breast cancer cells. Our findings show that 4EBP1 is a critical protein for luminal breast cancer cell proliferation regardless of amplicon and/or ER status. However, shRNA mediated knockdown of 4EBP1 in non-transformed mammary epithelial cells did not affect proliferation. It is possible that complete knock-out of 4EBP1 in non-tumorigenic breast epithelial cells could affect their proliferative capacity, but our results indicate that the changes in 4EBP1 expression in luminal breast cancer cells achieved by shRNA knockdown is sufficient to profoundly affect their proliferative capacity. Consistent with the idea that 4EBP1 has a potential role in regulating ERα expression, as well as a potential role outside of ERα regulation, we found that downregulation of 4EBP1 reduces not only ERα expression but also affects Cyclin D1 expression and p27 expression. These observations are consistent with the reduced proliferation and cell cycle arrest phenotypes that we report in our present study. There is no indication that Cyclin D1 or p27 levels would change in non-transformed cells because cell proliferation was not compromised with 4EBP1 knockdown in these models. Future studies should further explore the relationship between 4EBP1 and Cyclin D1 in cancer cells and non-transformed cells. There is a consistently demonstrated occurrence between co-amplification of genomic loci harboring 4EBP1 (*EIF4EBP1*) and Cyclin D1 (*CCND1*) in breast cancer patients such as the recent report by *Giltnane and colleagues* [27], so further studies should assess how these two oncogenes together can influence cell cycle states, meiotic progression, and the regulation of aneuploidy. Because 4EBP1 is required for coupling mTORC1 signaling to Cyclin D1 expression [101] and translational inhibition can result in the loss of cell cycle regulators like the D-cyclins [105], we plan to determine the predictive value of 4EBP1 levels to CDK inhibition in breast tumors, especially in the context of dual inhibition with PI3K/AKT/mTOR inhibitors.

Amplification of *EIF4EBP1* leads to increased 4EBP1 expression and phosphorylation suggesting that mechanisms are in place to promote 4EBP1 mediated translation and post-translational regulation during breast cancer initiation and progression. Consequently, targeting of 4EBP1 either directly or via inhibition of mTOR could relieve repressive effects of phosphorylated 4EBP1 on translation as well as any capacity of 4EBP1 to stabilize mTORC1 [106] or other proteins like p21 [107]. Several Phase II clinical trials have evaluated use of mTOR inhibitors for ER+ breast cancer [108–110]. While promising, results from trials in patients with ER+ breast cancer who experience aromatase inhibitor failure were only somewhat successful [108]. However, a current clinical trial is underway to determine if the phosphorylation status of 4EBP1 can be used to predict everolimus response in breast tumors (NCT00855114).

Direct targeting of 4EBP1 or targeting of multiple upstream kinases that target 4EBP1 may provide additional benefit. Recently, several kinases were identified to phosphorylate 4EBP1 in both mTOR dependent as well as independent manners [41, 42]. Of note, GSK3β phosphorylation of 4EBP1 plays a similar role as mTOR, whereby phosphorylation decreases 4EBP1 association with eIF4E [50]. Contrary to this observation, CDK1 is a mitotic kinase that also phosphorylates 4EBP1 [67, 70]. However, phosphorylation by CDK1 does not alter the cap-dependent translation functions of 4EBP1. Interestingly, a phospho-deficient mutant of 4EBP1 that is resistant to phosphorylation by CDK1 partially reverses rodent cell transformation. It is suggested that 4EBP1 phosphorylation by CDK1 could result in a gain of function, which opposes the canonical form of regulation set forth by studies evaluating mTOR-mediated inhibition of 4EBP1 through phosphorylation. Regulation of phosphorylated 4EBP1 especially the intertwined dynamics between CDK1 and mTOR should be further explored, as CDK1 can phosphorylate mTOR and co-localize with phosphorylated 4EBP1 [59]. Whether the distinct effects of the different phosphorylation states of 4EBP1, determined by distinct phosphorylation events driven by individual kinases, affects 4EBP1’s ability to drive breast cancer progression or endocrine resistance would be of significant interest for future studies particular in the context of therapeutic interventions.

## Conclusions

EIF4EBP1 is a candidate oncogene in breast cancer because it is commonly amplified and overexpressed, and is part of a genomic region that, when amplified, confers poor prognosis for patients. Overexpression of 4EBP1 drives proliferation of luminal breast cancer cells by mechanisms involving cell cycle regulators such as cyclin D1 and the cdk inhibitor p27. In some cells, 4EBP1 phosphorylation occurs with high level activity of the mTORC pathway, which also is common in estrogen-receptor positive breast cancer, and indeed, knockdown of EIF4EBP1 results in reduced expression of ERα. Based on these results, we conclude that 4EBP1, and particularly phosphorylated 4EBP1 plays a dominant role in breast cancer by mechanisms distinct from its role in regulating cap-dependent translation.

## Additional files

Additional file 1: **Figure S1** 4EBP1 knockdown inhibits proliferation of MCF7 and T47D breast cancer cells. (**a**) Western blot of 4EBP1 in MCF7 cells and (**b**) T47D cells engineered with either control shRNA to *lacZ* or two individual shRNAs to *EIF4EBP1* (4EBP sh_1 or sh_2). (**c**) Cell proliferation was assessed in MCF7 and (**d**) T47D control and *EIF4EBP1* knockdown cells on day 1 and day 4 in culture. Error bars represent standard deviation among replicates and *p*-values represent the comparison between each corresponding group. (TIF 2538 kb)

Additional file 2: **Figure S2** 4EBP1 knockdown slows proliferation of SUM-229 and SUM-149 breast cancer cells. (**a**) Western blot of 4EBP1 in SUM-229 cells and (**b**) SUM-149 cells engineered with either control shRNA to *lacZ* or two individual shRNAs to *EIF4EBP1* (4EBP sh_1 or sh_2). (**c**) Cell proliferation was assessed in SUM-229 and (**d**) SUM-149 control and *EIF4EBP1* knockdown cells on day 1 and day 4 in culture. Error bars represent standard deviation among replicates and signficance is shown between each corresponding group. (TIF 2688 kb)

Additional file 3: **GrowthResults_2–15-19.xlsx** Results and statistical analysis of experiments in which EIF4EBP1 was knocked down in three breast cancer cell lines. (XLSX 16 kb)

## Abbreviations

4EBP1: Eukaryotic Initiation Factor 4E-Binding Protein
EIF4EBP1: Eukaryotic Initiation Factor 4E-Binding Protein
ER: Estrogen receptor alpha
RFS: Relapse Free Survival
