# Supplementary figures and images for: Eukaryotic initiation factor 4E-binding protein as an oncogene in breast cancer
Source: BMC Cancer. 2019 May 23;19:491. doi: 10.1186/s12885-019-5667-4 (PMC6533768; doi:10.1186/s12885-019-5667-4)

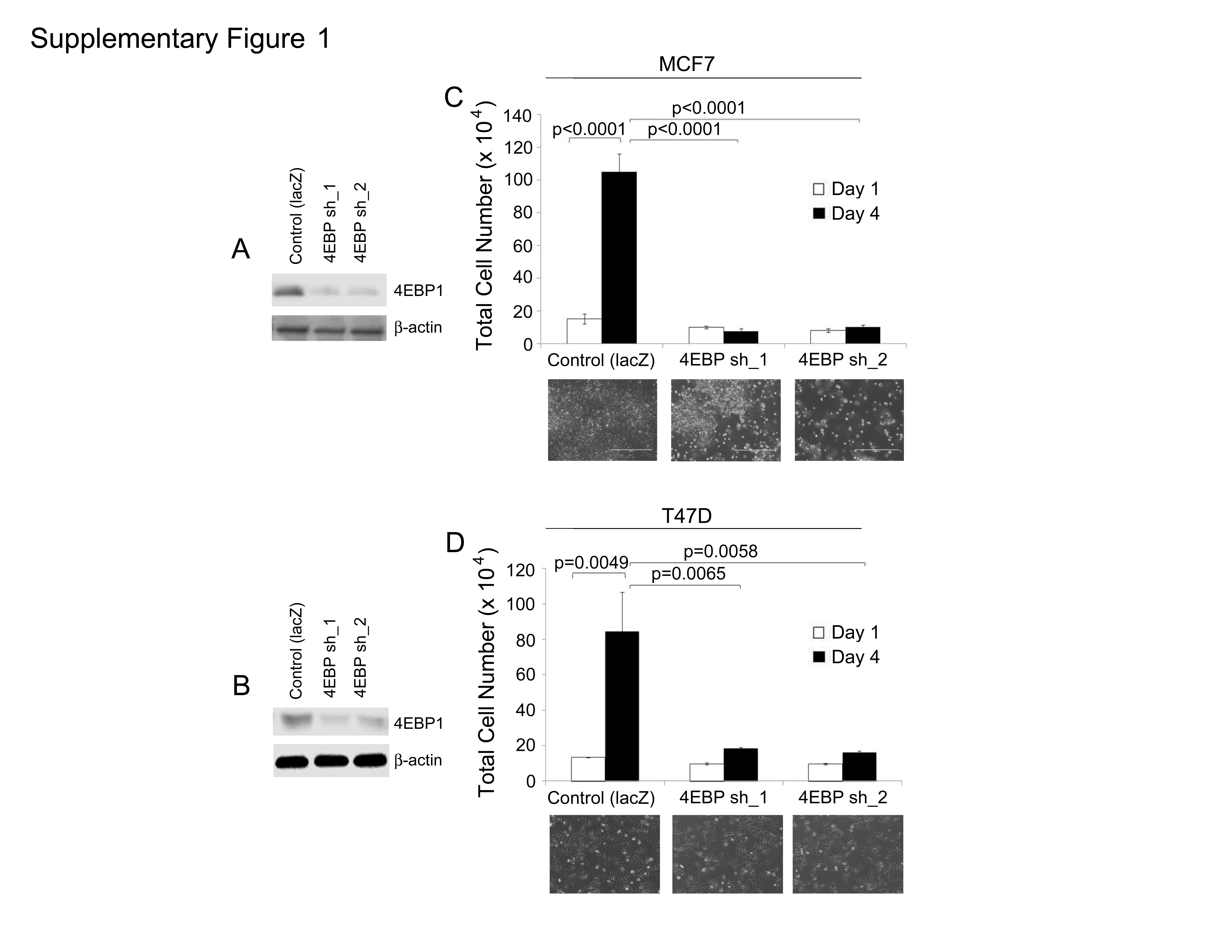

Supplement: Supplementary file 1 — Figure S1 4EBP1 knockdown inhibits proliferation of MCF7 and T47D breast cancer cells. (a) Western blot of 4EBP1 in MCF7 cells and (b) T47D cells engineered with either control shRNA to lacZ or two individual shRNAs to EIF4EBP1 (4EBP sh_1 or sh_2). (c) Cell proliferation was assessed in MCF7 and (d) T47D control and EIF4EBP1 knockdown cells on day 1 and day 4 in culture. Error bars represent standard deviation among replicates and p-values represent the comparison between each corresponding group. (TIF 2538 kb) [file 12885_2019_5667_MOESM1_ESM.tif]

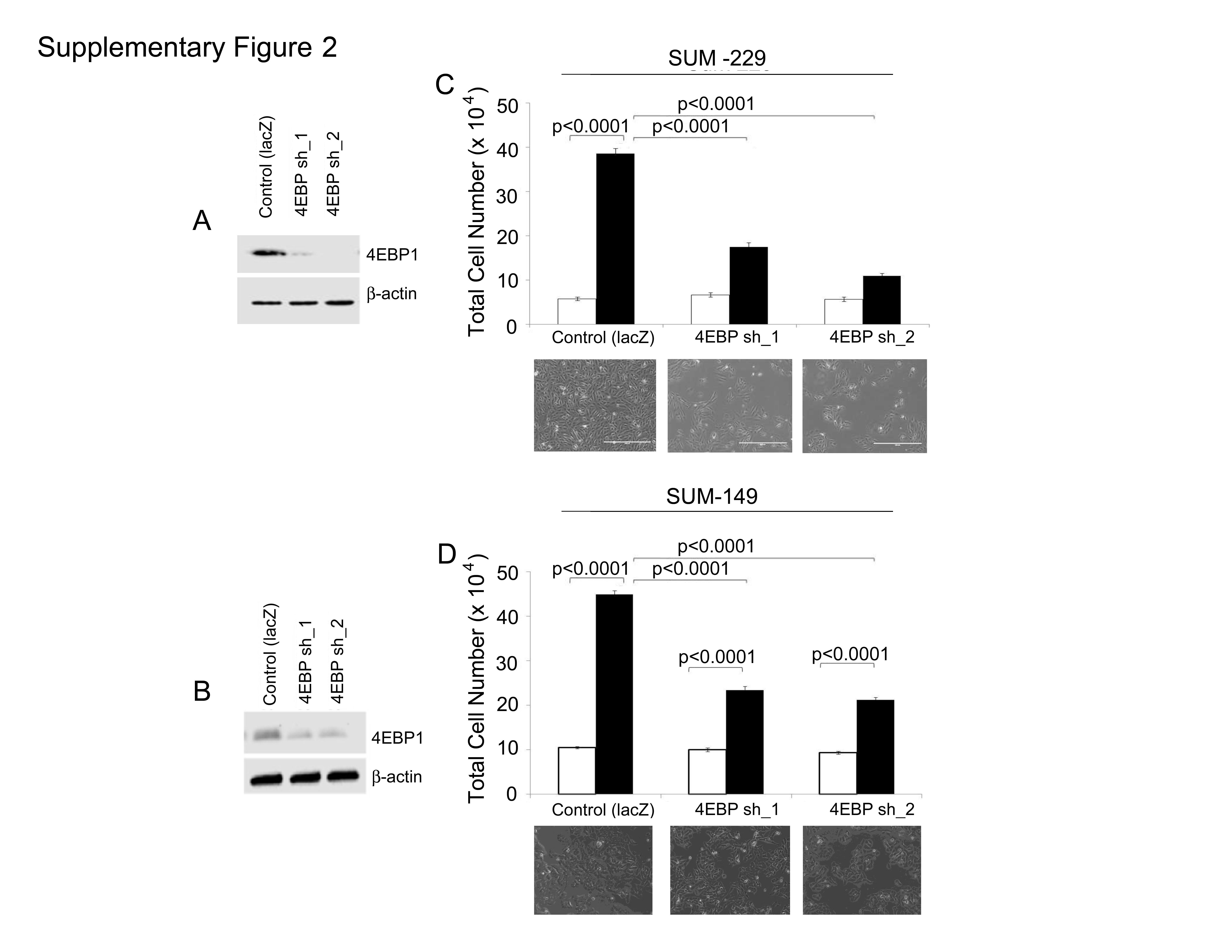

Supplement: Supplementary file 2 — Figure S2 4EBP1 knockdown slows proliferation of SUM-229 and SUM-149 breast cancer cells. (a) Western blot of 4EBP1 in SUM-229 cells and (b) SUM-149 cells engineered with either control shRNA to lacZ or two individual shRNAs to EIF4EBP1 (4EBP sh_1 or sh_2). (c) Cell proliferation was assessed in SUM-229 and (d) SUM-149 control and EIF4EBP1 knockdown cells on day 1 and day 4 in culture. Error bars represent standard deviation among replicates and signficance is shown between each corresponding group. (TIF 2688 kb) [file 12885_2019_5667_MOESM2_ESM.tif]
